# Can balancing selection on MHC loci counteract genetic drift in small fragmented populations of black grouse?

**DOI:** 10.1002/ece3.86

**Published:** 2012-02

**Authors:** Tanja M Strand, Gernot Segelbacher, María Quintela, Lingyun Xiao, Tomas Axelsson, Jacob Höglund

**Affiliations:** 1 Population Biology and Conservation Biology, Department of Ecology and Genetics, Evolutionary Biology Centre, Uppsala UniversityNorbyvägen 18D, SE-752 36 Uppsala, Sweden; 2 Department Wildlife Ecology and Management, University FreiburgTennenbacher Str. 4, D-79106 Freiburg, Germany; 3 Faculty of Science, Department of Animal Biology, Plant Biology and Ecology, University of A CoruñaCampus da Zapateira, E-15171 A Coruña, Spain; 4 Department of Medical Sciences, Molecular Medicine, Uppsala UniversityAkademiska sjukhuset ing. 70, SE-751 85 Uppsala, Sweden

**Keywords:** Fragmentation, genetic drift, MHC, population isolation, SNP

## Abstract

The ability of natural populations to adapt to new environmental conditions is crucial for their survival and partly determined by the standing genetic variation in each population. Populations with higher genetic diversity are more likely to contain individuals that are better adapted to new circumstances than populations with lower genetic diversity. Here, we use both neutral and major histocompatibility complex (MHC) markers to test whether small and highly fragmented populations hold lower genetic diversity than large ones. We use black grouse as it is distributed across Europe and found in populations with varying degrees of isolation and size. We sampled 11 different populations; five continuous, three isolated, and three small and isolated. We tested patterns of genetic variation in these populations using three different types of genetic markers: nine microsatellites and 21 single nucleotide polymorphisms (SNPs) which both were found to be neutral, and two functional MHC genes that are presumably under selection. The small isolated populations displayed significantly lower neutral genetic diversity compared to continuous populations. A similar trend, but not as pronounced, was found for genotypes at MHC class II loci. Populations were less divergent at MHC genes compared to neutral markers. Measures of genetic diversity and population genetic structure were positively correlated among microsatellites and SNPs, but none of them were correlated to MHC when comparing all populations. Our results suggest that balancing selection at MHC loci does not counteract the power of genetic drift when populations get small and fragmented.

## Introduction

Human-induced habitat loss and habitat fragmentation are arguably the greatest threats to the survival and persistence of natural populations (Morris and Doak 2002). These processes lead to smaller and more isolated populations that face increased risk of extinction both through ecological factors, such as demographic stochastic events, and adverse genetic effects, such as increased levels of inbreeding and genetic drift (Höglund 2009a). The relative roles of ecological versus genetic factors in local extinction have been debated and it has been argued that most populations may go extinct for ecological reasons before genetic factors have any chance to impact on them (Lande 1988; Caughley 1994). However, meta-analyses have highlighted that genetic factors do impact threatened species before extinction (Reed and Frankham 2003; Spielman et al. 2004b). Thus, it is possible that threatened taxa may have lost the ability to adapt to changing environmental conditions and this might be one reason for why such species are more prone to extinction (Ellstrand and Elam 1993; Amos and Balmford 2001). Host genetic diversity may also buffer against diseases (Altizer et al. 2003; Spielman et al. 2004a). However, it is not known whether loss of genetic diversity is important across the whole genome (an heterosis effect) or at key loci of large effect (due to deleterious recessives) (Balloux et al. 2004).

Given that neutral genetic variation may often provide an incomplete picture of the evolutionary potential of populations (e.g., Bekessy et al. 2003; Hoffmann et al. 2003), it has been suggested that it is important to monitor adaptive genetic diversity in natural populations. Adaptive diversity is defined as “genetic variation that produces an advantage in fitness” (Hedrick 2001). Genes suitable to be used as proxy for adaptive genetic diversity should be highly variable. Major histocompatibility complex (MHC) genes in vertebrates play an important role in the immune defense and are subjected to balancing selection (Garrigan et al. 2003; Piertney and Oliver 2006). Numerous associations have been found between MHC genotypes and pathogen resistance (Meyer–Lucht and Sommer 2005; Westerdahl et al. 2005; Bonneaud et al. 2006; Oppelt et al. 2010) and MHC diversity is related to survival in birds (Bonneaud et al. 2004; Brouwer et al. 2010; Worley et al. 2010). These MHC genes, which is one of the most suitable candidates for studies of adaptive genetic diversity (Piertney and Oliver 2006) and MHC variation, have become increasingly important for monitoring endangered species such as giant panda (Wan et al. 2006), tigers (Pokorny et al. 2010), and African wild dogs (Marsden et al. 2009).

**Figure 1:**
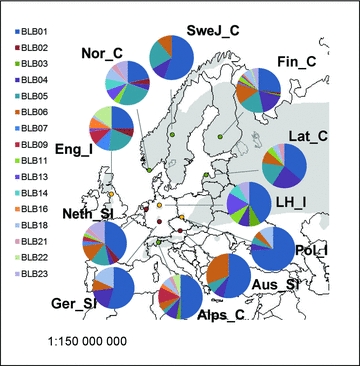
Map of Europe showing pie charts for each population with frequency of MHC class II alleles. The black grouse distribution range is shown in grey (distribution map from Storch 2007). The colors of the dots (and the names) indicate the population category; green for continuous (_C), yellow for isolated (_I), and red for small isolated populations (_SI). Population code in Table 1.

The black grouse (*Tetrao tetrix*) is an ecologically well-studied species. Within Europe, it is a flagship and umbrella species for the conservation of open woodlands, heather moors, and bogs. It has a wide distribution from the United Kingdom in the West, to the mountains bordering the People's Republic of China and North Korea in the East (the European range is displayed in Fig. 1). Populations in western and central Europe have rapidly decreased since the 1970s and many small ones have become extinct (Klaus et al. 1990). The major cause of this decline is habitat degradation and deterioration due to intensified human land-use (e.g., forest plantations and drainage of moorlands). Habitat preservation is thus considered to be crucial for the survival of remaining black grouse populations. The global Red List status is “least concern” but, in fact, 14 European countries have red-listed the species (Storch 2007). Black grouse populations in Europe vary greatly in population status and size ranging from only a few displaying cocks in several small isolated populations in central Europe to a more continuous distribution in Fenno–Scandia and Russia (Bauer et al. 2005; Höglund 2009b). Populations can be isolated several hundred kilometres from each other making contemporary exchange between such populations through immigration very unlikely (Höglund et al. 2007).

The aim of this study was to investigate if (1) small isolated populations have lower microsatellite, SNP, and MHC genetic diversity than large and continuous populations. We additionally tested (2) if the genetic diversity and population structure were different for MHC than for SNPs and microsatellites and (3) explored correlations between SNP and microsatellite markers.

## Material and Methods

### Sampling

We sampled 320 black grouse from 11 European locations (hereafter referred to as populations) that were grouped into different categories according to their status (Table 1). The populations were divided into three categories: firstly, “continuous” meaning large continuous populations of at least 1000 individuals; secondly, “isolated” meaning populations of less than 300 individuals well separated (>300 km apart) from other populations; and thirdly, “small isolated” consisting of less than 50 individuals. Isolation can be assumed from the small dispersal distances reported for this species (∼ 25km, Storch and Segelbacher 2000). The approximated population sizes are based on a survey from Birdlife International (2004) and personal communication with conservation practitioners and other black grouse researchers. The samples were collected between 1989 and 2008. As we analyze categories rather than individual populations, we minimize possible temporal sampling effects on genetic variation. We used different sources for DNA such as tissue, blood, molted feathers (Lüneburger Heide, Sudety Mountains, Rhön, Waldviertel, and Northern Pennines), or feathers plucked from shot birds (Alps). Birds from the study site in Finland were captured and circa 1-mL blood samples were drawn from the brachial wing vein with a syringe using a heparinized needle. Red blood cells were separated from plasma by centrifuging at 12,000 rpm for 5 min, and stored at 75% alcohol at +4°C until DNA extraction. Samples from Latvia, Sweden, and Norway were obtained from muscle tissue from harvested individuals. The Dutch black grouse samples included a mixture of feathers, hatched eggshells, and tissue from carcasses. DNA extracted from feathers may be of poor quality (e.g., Horváth et al. 2005; Johansson et al. in press). However, DNA quality was comparable among samples, as only feather DNA extracts that have been shown to yield DNA for reliable genotyping have been used in addition to other tissue samples. DNA was extracted either using a salt-extraction procedure (Paxton et al. 1996) or the Qiagen DNeasy Blood & Tissue isolation kit (Qiagen Inc., Hilden, Germany) according to the manufacturer's instructions. To avoid contamination, DNA extractions, pre-PCR and post-PCR pipetting, were carried out in different rooms, and the equipment was sterilized by using UV radiation. Negative controls were included throughout.

**Table 1:** Population categories, locations, and time of sampling. The year marked with “^1^” was analyzed for MHC and SNP data only, and the year marked with “^2^” for microsatellites. *N* is the approximate sample size in each population

Population category	Population	Code	Country	Year of sampling
Continuous (*N*≍>1000)	Jyväskylä	Fin_C	Finland	2001^1^, 1989–95^2^
	Kristiansand	Nor_C	Norway	1990–1991
	Jämtland	SweJ_C	Sweden	2007
	Latvia	Lat_C	Latvia	2002–2003
	Alps	Alps_C	Switzerland	2005
Isolated (*N* <300)	Northern Pennines	Eng_I	UK	2000–2006
	Lüneburger Heide	LH_I	Germany	1994–2008
	Sudety Mountains	Pol_I	Poland	2007–2008
Small isolated (*N*≍30)	Sallandse Heuvelrug	Neth_SI	Netherlands	2003–2006
	Waldviertel	Aus_SI	Austria	2002–2003
	Rhön	Ger_SI	Germany	1992,93,95,2003

Of the 320 individuals included in this analysis, we obtained microsatellite genotypes for 307 individuals, SNP genotypes for 229 individuals and scored allelic variation at two (or three) MHC class IIB loci for 164 individuals. Not all individuals where we obtained full microsatellite genotypes could be scored for MHC or SNPs, and in a few cases, we were able to amplify the SNPs but were not able to genotype the individual at the microsatellite loci (see Table 2).

### Microsatellite genotyping

DNA samples were genotyped at nine microsatellites: TUT1, TUT3, TUT4, BG10, BG12, BG15, BG16, BG18, and BG19 (Segelbacher et al. 2000; Piertney and Höglund 2001). The success of DNA extraction was tested by amplifying one microsatellite locus (TUT1) that was electrophoresed in a 1.2% agarose minigel; thus only samples that yielded a product of the appropriate size were retained for subsequent genotyping. All samples were genotyped at least twice to ensure the reliability of the genotypes (see also Segelbacher et al. 2008). PCR amplifications were performed in a total volume of 10 µl using an Eppendorf Gradient thermal cycler (Eppendorf, Hamburg, Germany). Each reaction mixture contained 2 µl of DNA extract, 2.5 mM MgCl_2_, and 1 µl of Eppendorf PCR buffer (Eppendorf), 0.2 mM of each nucleotide, 0.5 mM of each primer, and 0.5 units *Taq* polymerase (Eppendorf HotmasterTaq; Eppendorf). PCR profiles comprised 35 cycles of 30-sec denaturation at 94°C, 30-sec annealing at 54°C (for BG loci) or 60°C (for TUT loci), and 30-sec extension at 68°C. PCR fragments were resolved by electrophoresis on an ABI 377 automated sequencer (Applied Biosystems, Foster City, California, USA). Allele sizes were determined by reference to two standard samples run simultaneously: (1) the ROX 350 Ladder (Applied Biosystems); (2) a black grouse individual previously genotyped at the same loci. To rule out contamination of samples with exogenous DNA or PCR products, tubes with water instead of sample/template were included in the DNA extraction and PCR amplification procedure as negative control.

### SNP identification and genotyping

Twenty-four protein-coding genes of length ranging between 324 and 809 bps were amplified in 15 µl reactions in an Applied Biosystems Gene Amp PCR Systems 2700 thermal cycler. Individual mixes contained approximately 40 ng DNA template, 1× PCR buffer, 1.5–2.5 mM MgCl_2_, 1× GC, 0.1 mM dNTP (Fermentas, St. Leon-Rot, Germany), 0.25 µM of each primer, and 0.375 U FastStart Taq Roche polymerase (Roche Diagnostics Scandinavia AB, Stockholm, Sweden). The protocol was the same as used for a study on the closely related willow/red grouse (*Lagopus lagopus*). The names and localization of the genes on the chicken (*Gallus gallus*) chromosomes, the sequences of the primers and the PCR profiles are compiled in Berlin et al. (2008). For automated sequencing, PCR products were purified with ExoSAP-IT (USB Corporation, Cleveland, Ohio, USA) and sequenced on a MegaBACE™ 1000 capillary instrument (GE Healthcare, Uppsala, Sweden). Sequence tracings were analyzed using the Sequencher 4.0.5 software (Gene Codes). A putative SNP was considered true when PHRED quality scores of the different variants exceeded 25. Individuals from the large continuous populations (that should retain more genetic variability) such as Finland or Norway were used in the SNP detection process, thus obtaining a total of 34 SNPs (ranging from none to six per exon) across 12,781 bp. Primers were designed for those 34 SNPs and multiplex genotyping was performed using the GenomeLab SNPstream system (Beckman Coulter, Fullerton, California, USA) (Bell et al. 2002) available at the SNP & SEQTechnology Platform at Uppsala University (http://www.genotyping.se). Twenty-one of the selected 34 SNPs (of which a majority were synonymous) were successfully amplified at multiplex yielding one SNP for every 376 bp in the genes bcl2, BRIP, CAAX, EPN, GCM, KELCH, LEPR, MBL, MICRO, NGF, PKP4, PROOPIO, TRANS, and YTH.

### MHC class II genotyping

Using the black grouse MHC class II B *(BLB)* primer pair RNAF1a (5’-GACAGCGAAGTGGGGAAATA-3’) and RNAR1a (5’-CGCTCCTCTGCACCGTGA-3’), we amplified *BLB* alleles from gDNA, for details see Strand et al. (2007). We have earlier demonstrated that this primer pair amplifies expressed *BLB* loci in black grouse and that this species has two to three BLB loci (Strand et al. 2007). Although we cannot completely rule out the possibility of copy number variation (see e.g., Eimes et al. 2011) in and among black grouse populations, further sequencing suggest that two BLB loci in black grouse is standard (T. Strand, B. Wang, Y. Meyer-Lucht, and J. Höglund, unpubl. data). The RNAF1a/RNAR1a primer pair amplifies both loci simultaneously and yields PCR-products that starts at the 108th base pair in exon 2 and stops at the 270th (the last) bp of exon 2 (46% of the exon covering about 2/3 of the peptide binding sites). For the MHC genotyping, we have used reference strand-mediated conformational analysis (RSCA; Arguello et al. 1998), for details of our RSCA method, see Strand and Höglund (2011). New RSCA peaks for this study were detected and to identify their sequence identification, in total 19 individuals across populations were cloned and sequenced. The RSCA dataset was randomized blindly before performing the RSCA scoring, so that the identity of population category was unknown.

### Data analysis

We tested for outliers from neutrality in microsatellites and SNP markers using two different approaches. In the case of microsatellites, we first used the hierarchical Bayesian method described in Beaumont and Balding (2004) as implemented in BayeScan 2.01 software (Foll and Gaggiotti 2008) that estimates population-specific FST coefficients and uses a cutoff based on the mode of the posterior distribution. The program was run by setting sample size to 10,000, burn in to 100,000, and the thinning interval to 50 as suggested by Foll and Gaggiotti (2008), resulting in a total chain length of 600,000 iterations. Secondly, we used the Beaumont and Nichols (1996) Fdist approach implemented in LOSITAN (Antao et al. 2008) simulating the neutral distribution of FST with 100,000 iterations at a significance *P-*value of 0.005. Runs were performed using the two possible mutation models: the stepwise mutation model and the infinite allele model. For SNPs, we only used BayeScan 2.01 software that implements a new function for outlier detection in this type of markers. As we did not find any locus deviating from neutrality for either microsatellites or SNPs, we hereafter treat them as presumably neutral.

The Software GIMLET (Valière 2002) was used to assess the reliability of identifying individuals (from the molted feather samples) and to estimate error rates due to microsatellite allelic drop out. For microsatellites, the genotype distribution of each locus in each subpopulation was compared with the expected Hardy–Weinberg distribution using the program FSTAT 2.9.3 (Goudet 1995) as was the genotypic disequilibrium among loci. DNA quality and genotyping in the dataset was reliable across samples and individuals as we did not detect any genotyping errors or dropout rates for microsatellites within the analyzed dataset. For microsatellites, the allele frequencies, the estimates of within-population call diversity (observed number of alleles and heterozygosity), and among population diversity (Weir and Cockerham 1984) analogue to Wright's F_ST_ were calculated using FSTAT 2.9.3 (Goudet 1995) and GENETIX (Belkhir et al. 2004). Allelic richness (AR) was also calculated in FSTAT, taken the smallest sampled population into account. For SNPs, the expected Hardy–Weinberg distribution was calculated using the program Genepop (Raymond and Rousset 1995). The SNP genotypic disequilibrium among loci, summary statistics, and F_ST_ were calculated in GenAlEx (Peakall and Smouse 2006). Differences between diversity measures among population categories were tested with analysis of variance (ANOVA) in R v2.13.1 and post hoc tests were performed with Tukey's HSD, also in R.

MHC alleles were aligned and edited with the CodonCodeAligner software version 3.7.1 (LI-COR, Inc.). In previous MHC studies, generally only alleles present in two independent PCR reactions are regarded as confirmed (Westerdahl et al. 2004). The nucleotide sequences of the new confirmed alleles in this study were deposited in GenBank (see Data Archiving section). We cannot assign MHC class II alleles to loci, so therefore it was not possible to calculate MHC heterozygosity values. Genetic diversity for MHC was instead calculated using average percentage difference of alleles in populations (APD), theta *k* (index of AR), nucleotide diversity Pi, total number of different MHC alleles in a population divided by sample size (MHC/pop), and mean number of MHC alleles per individual (MHC/ind). MHC Pi and theta *k* was calculated in Arlequin 3.5.1.2 (Excoffier et al. 2005). APD measures the average percentage of alleles that differ among individuals (see Miller et al. 2010), and we calculated mean APD in MATLAB version 7.11. (Natick, MA; The MathWorks Inc., 2010). Differences between MHC diversity measures among population categories were tested with ANOVA in R. Pairwise MHC F_ST_ were calculated in Arlequin by entering the nucleotide sequence of the MHC allele and number of individuals with that allele in each population as haplotype data.

F_ST_ is argued to be appropriate to measure for biallelic markers such as SNPs but the value of F_ST_ may be affected by highly variable markers. For this reason, we compared classical F_ST_ with Jost's D (D_EST_) (Jost 2008) for all our three types of markers. D_EST_ were calculated for microsatellites and SNP markers using the online program SMOGD v1.2.5 (1000 bootstraps) (Crawford 2010) and for MHC in the program SPADE (version Feb 2009) (Chao and Shen 2010) (10,000 bootstrap). Microsatellite pairwise D_EST_ and F_ST_ were highly significantly correlated (*r*_M_ = 0.89, *P* < 0.001) also when correcting for geographic pairwise distance (partial *r*_M_ = 0.90, *P* < 0.001). As for microsatellites, SNP pairwise D_EST_ and F_ST_ were highly significantly correlated (*r*_M_ = 0.83, *P* < 0.001, partial *r*_M_ = 0.84, *P* < 0.001). MHC pairwise D_EST_ and F_ST_ were also highly significantly correlated (*r*_M_ = 0.86, *P* < 0.001, partial *r*_M_ = 0.87, *P* < 0.001). Since the fixation index F_ST_ and the differentiation index D_EST_ were strongly significantly correlated for all marker types, we decided to only report F_ST_ values in the result section (but see Table S1 for D_EST_ values).

The correlation between pairwise estimates of F_ST_/(1 – F_ST_) (Rousset 1997) for different markers was evaluated by performing Mantel tests and partial Mantel (Pearson statistics) tests (controlling for geographical distance) using the Vegan package v1.17–11 (Oksanen et al. 2009) in R (10,000 matrix permutations). Isolation by distance was tested by comparing F_ST_/(1 – F_ST_) and natural logarithm of distance in kilometre between populations in Mantel tests (and correcting for neutral markers when testing MHC using partial Mantel tests) in the Vegan package in R (10,000 matrix permutations). All statistical tests of multiple comparisons were Bonferroni corrected (correlation tests and pair-wise F_ST_ analyses).

## Results

**Figure 2:**
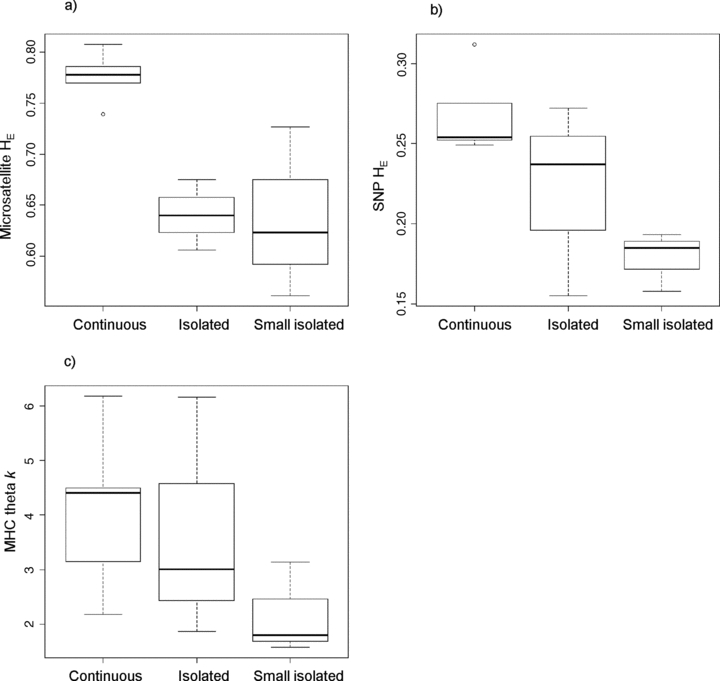
Genetic variation comparing population categories continuous (*n* = 5), isolated (*n* = 3), and small isolated (*n* = 3) for (a) microsatellite expected heterozygosity (H_E_), (b) SNP expected heterozygosity (H_E_), and (c) MHC theta *k*.

At the nine microsatellite loci, the genotype distribution deviated from Hardy–Weinberg equilibrium (HWE) in one continuous population (Fin_C), one isolated population (Pol_I), and three small isolated populations (Neth_SI, Ger_SI, and Aus_SI). We could not detect linkage disequilibrium between loci or indication of null alleles for any locus. Values of microsatellite expected heterozygosity (H_E_) varied considerable, between 0.56 and 0.81 (Table 2; Fig. 2a). AR varied between 3.43 and 5.92 between the populations (Fig. S1a).

**Table 2:** Summary statistics for microsatellites, SNPs, and MHC showing number of scored individuals (*N*), expected heterozygosity (H_E_), allelic richness (AR), polymorphic loci in percentage (Poly), mean number of MHC alleles per individual (MHC /ind), total number of different MHC alleles in pop/sample size (MHC/pop), mean average percentage difference among individuals (APD), Pi (mean number of pairwise differences between all pairs of alleles in the population), and theta *k* (index of allelic richness)

	Microsatellites			SNPs			MHC					
			
Code	*N*	He	AR	*N*	He	Poly	*N*	MHC/ind	MHC/pop	APD	Pi	theta *k*
Fin_C	57	0.78	5.76	22	0.31	85.71	29	2.97	0.48	53.01	13.96	4.50
Nor_C	31	0.74	5.32	21	0.25	76.19	11	2.09	0.91	75.31	12.68	6.18
SweJ_C	14	0.79	5.58	14	0.25	71.43	6	1.50	0.67	52.22	12.61	2.18
Lat_C	13	0.81	5.92	12	0.27	76.19	9	2.44	0.78	49.10	13.34	3.14
Alps_C	57	0.77	5.44	25	0.25	76.19	22	1.55	0.45	52.28	11.07	4.40
Eng_I	21	0.61	3.43	20	0.15	57.14	18	2.67	0.50	63.54	12.49	3.00
LH_I	24	0.64	4.83	36	0.24	76.19	6	2.00	1.17	54.76	8.83	6.15
Pol_I	23	0.68	4.31	26	0.27	76.19	14	1.36	0.36	21.43	7.59	1.86
Neth_SI	31	0.56	3.49	20	0.19	47.62	34	2.53	0.32	46.90	13.09	3.13
Aus_SI	14	0.62	4.09	17	0.18	57.14	8	1.63	0.50	41.67	13.10	1.57
Ger_SI	22	0.73	5.05	16	0.16	47.62	7	1.57	0.57	50.00	10.40	1.80

In the SNP dataset, two markers deviated from HWE: locus BRIP-172 in one isolated population (LH_I) and CAAX-200 in one small isolated population (Aus_SI). Likewise, linkage disequilibrium was detected for the loci pair GCM-273 and GCM-317 and for the loci pair MBL-442 and MBL-610. The amount of H_E_ varied across populations between 0.15 and 0.31 (Table 2; Fig. 2b) for the 229 SNP-genotyped individuals. We also found a large difference between populations for percentage polymorphic SNP loci (from 47.62 to 85.71).

We found one to five MHC BLB alleles per individual by RSCA (mean number of alleles per individual across populations was 2.21, *SD* 1.13) (Table 2). The RSCA scored one allele in 53 individuals, two alleles in 53 individuals, three alleles in 35, four alleles in 16, and five alleles in seven individuals. MHC allele “Tete BLB01” was the most frequent among individuals; 135 of 164 (82%) had this allele (Fig. S2). We did not observe any private MHC alleles in the populations (Fig. 1). To determine the allele identity of the RSCA peaks, we cloned 19 individuals across populations and found 16 unique and confirmed MHC class II B alleles, eight of which were not published before. The cloned individuals possessed between one and four confirmed MHC alleles, all with unique amino acid sequences. Thus, it is possible that RSCA overestimates the number of alleles. Seven of these 19 cloned individuals were from continuous populations (five Fin_C and two Nor_C), five from an isolated population (Eng_I), and seven from a small isolated population (Neth_SI).

### Neutral and MHC genetic diversity in populations of varying size and isolation degree

There were significant differences between the black grouse population categories continuous, isolated, and small isolated in microsatellite heterozygosity (H_E_) and allelic richness (AR) (F_2,8_ = 10.9, *P* = 0.0052; F_2,8_ = 8.7, *P* = 0.0098) (Figs. 2a and S1a). Both small isolated populations and isolated populations had significantly lower H_E_ and AR than the continuous populations (Tukey's HSD, adj. *P* < 0.05 for both). The two measures of microsatellite genetic diversity, H_E_ and AR were significantly correlated (*r* = 0.95, *df* = 9, *P* < 0.001, *n* = 11) (Table 3).

**Table 3:** Correlation tests with Pearson's product-moment correlation. The *P* -values are given below the diagonal, with values significant after Bonferroni correction in bold. Above the diagonal are the *r* -values. Microsatellite- (MS) and SNP-expected heterozygosity (H_E_) are reported as well as allelic richness (AR), polymorphic loci in percentage (Poly), mean average percentage difference among individuals (APD), Pi (mean number of pairwise differences between all pairs of alleles in the population), theta *k* (index of allelic richness), mean number of MHC alleles per individual (MHC /ind), and total number of different MHC alleles in pop/sample size (MHC/pop)

	MS He	MS AR	SNP He	SNP Poly	MHC APD	MHC Pi	theta *k*	MHC/ind	MHC/pop
MS He		0.95	0.64	0.62	0.15	0.17	0.13	–0.11	0.21
MS AR	<0.001		0.70	0.68	0.20	0.14	0.31	–0.07	0.40
SNP He	<0.05	<0.05		0.92	–0.12	0.02	0.39	0.12	0.17
SNP Poly	<0.05	<0.05	<0.001		0.05	–0.09	0.53	0.08	0.36
MHC APD	0.66	0.55	0.73	0.88		0.48	0.64	0.43	0.51
MHC Pi	0.62	0.68	0.96	0.80	0.14		0.01	0.60	–0.14
theta *k*	0.71	0.36	0.24	0.09	<0.05	0.98		0.36	0.65
MHC/ind	0.75	0.83	0.74	0.82	0.19	0.05	0.28		0.01
MHC/pop	0.54	0.22	0.61	0.28	0.11	0.68	<0.05	0.97	

The SNP genetic diversity followed the same patterns as the microsatellites with lower genetic diversity in isolated and small isolated populations. There were significant differences between each of the population categories for both SNP H_E_ and percentage polymorphic SNP loci (F_2,8_ = 5.8, *P* = 0.028; F_2,8_ = 12.8, *P* = 0.0032) (Figs. 2b and S1b). Small isolated populations had significantly lower SNP H_E_ and polymorphic loci than continuous populations (Tukey's HSD, adj. *P* < 0.05 and *P* < 0.01). Interestingly, small isolated populations also had significantly lower SNP polymorphic loci than isolated populations (Tukey's HSD, adj. *P* < 0.05). The SNP diversity measures SNP H_E_ (Fig. 2b) and SNP polymorphic loci (Fig. S1b) were significantly correlated (Table 3).

For MHC theta *k*, there was a trend for MHC genetic diversity to be lower in small isolated populations compared to continuous populations (Fig. 2c). Consistently, there was a tendency for MHC/pop, MHC/ind, and MHC APD to display lower genetic diversity in small isolated populations compared to continuous populations (Fig. S1c–e). Despite this pattern, we did not find significant differences between the continuous, isolated, and small isolated population categories for any of the measures for MHC diversity (ANOVA data not shown, but see Table 2 for outliers possibly due to low population size). The measure MHC theta *k* was correlated to MHC APD and MHC/pop but not significantly so after Bonferroni correction (Table 3).

### Genetic diversity and population structure patterns in MHC compared to neutral markers

There were no correlations of any kind between MHC genetic diversity and neutral genetic diversity (Table 3).

**Figure 3:**
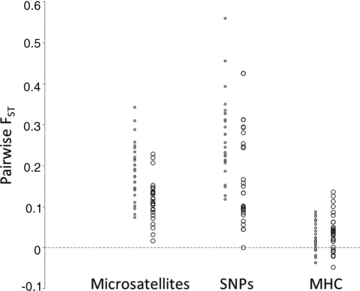
Plot over pairwise F_ST_ across populations for microsatellites, SNPs, and MHC. Each value is a data point for pairwise F_ST_ between two populations. All pairs are represented by open circles. Small circles are pairwise F_ST_ were at least one of the two populations is a small isolated population. The larger circles in a lane on the right side of the small circles are all other population pairs, including both isolated and continuous population pairs. The negative F_ST_ values (below the dotted line) should be interpreted as no differentiation between pairs.

The studied populations displayed different population structure for MHC as compared to neutral markers (Fig. 3). The global fixation index F_ST_ for microsatellite was 0.142 (95% CI, 0.122–0.166). Pairwise F_ST_ ranged from 0.0166 (SweJ_C–Lat_C) to 0.343 (Eng_I–Neth_SI) and all pairwise F_ST_ tests were significant (Table S1). Global SNP F_ST_ was 0.232 (*P* < 0.0001 after 10,000 permutations). Pairwise F_ST_ ranged from 0.001 (Fin_C–Lat_C) to 0.559 (Eng_I–Neth_SI) (Table S1). Forty-five of 55 population pairs were significant for F_ST_ (Table S1). As expected, for both microsatellites and SNPs, there is a trend for small isolated populations to be more genetically divergent than the other population categories (Fig. 3).

Contrary to microsatellites and SNPs, MHC F_ST_ did not show genetic differentiation between populations. The global MHC F_ST_ was 0.031 (*P* < 0.001 after 10,100 permutations). Pairwise F_ST_ ranged from zero for 15 population pairs (mainly pairs with SweJ_C and Ger_SI) to 0.136 (Eng_I–Pol_I) (Fig. 3; Table S1). All pairwise MHC F_ST_ tests for differentiation were nonsignificant.

For all markers, Eng_I was included in the pair that had the highest pairwise F_ST_. For both microsatellites and SNPs, Eng_I and Neth_SI was the most divergent pair, although they only differ in geographic distance at 630 km (compared to the longest distance, 2020 km, between the two populations separated most in space; Table S1).

Pairwise measurements between F_ST_/(1 – F_ST_) for MHC and microsatellites were not correlated (with or without correcting for distance). However, when removing the small isolated category from the dataset (keeping the isolated and continuous categories), MHC and microsatellite F_ST_/(1 – F_ST_) were significantly correlated (*r*_M_ = 0.63, P < 0.05) (also when corrected for distance (partial *r*_M_ = 0.62, *P* < 0.05). Pairwise measurements between F_ST_/(1 – F_ST_) for MHC and SNP were not significantly correlated neither when including all categories nor removing small isolated populations.

No isolation by distance (nor when removing small isolated populations from the analyses) was found for any of the three marker types when we compared the F_ST_/(1 – F_ST_) matrixes to the distance matrix (data not shown). However, when removing both small isolated and isolated populations from the analysis, leaving only continuous populations (n pop = 5), there was a significant isolation by distance for SNPs (*r*_M_ = 0.91, *P* < 0.01), but not for MHC or microsatellites.

### Correlations between SNPs and microsatellites

Pairwise estimates of F_ST_/(1 – F_ST_) for microsatellites and SNPs (Fig. S3a) were significantly correlated (*r*_M_ = 0.63, *P* < 0.01) also after controlling for geographical distance (partial *r*_M_ = 0.63, *P* < 0.01). Correlations were also observed between microsatellites (both H_E_ and AR) and SNPs (both H_E_ and Polymorphic loci) but not significantly so after Bonferroni correction (the H_E_ correlation visualized in Fig. S3b; see Table 3 for *r-* and *P*-values).

## Discussion

Small and highly fragmented populations often display lower neutral genetic diversity compared to larger populations (Höglund 2009a). Here, we used black grouse to test patterns of genetic variation at both neutral and MHC markers in a unique setting of different population categories, small isolated, isolated, and continuous, which vary in their degree of isolation and size. We have earlier reported lower neutral (microsatellite) genetic diversity in small isolated black grouse populations compared to larger populations (Höglund et al. 2007; Larsson et al. 2008). In this study, we further wanted to explore the reduction in genetic diversity, combining data from neutral microsatellite markers with genetic information derived from analysis of SNPs and MHC genes. Several authors point out the importance of using neutral sequence polymorphisms (such as SNPs) instead of microsatellites, when comparing neutral variation with MHC (e.g., Spurgin and Richardson 2010). It is argued that as both MHC and SNPs evolve through point mutations, this comparison would be more accurate than comparisons with microsatellites.

### MHC versus SNP and microsatellite genetic diversity

Small isolated populations of European black grouse showed significantly lower genetic diversity than continuous populations for both microsatellites and SNP markers (Fig. 2a and 2b). This result confirms earlier observations of decreased neutral microsatellite genetic diversity in small fragmented populations of black grouse compared to larger populations (Höglund et al. 2007; Larsson et al. 2008; Höglund et al. 2011).

Importantly, we found that small isolated populations have not only lower neutral but also lower MHC genetic diversity than large and continuous populations, although the latter is not as pronounced. Despite the parallel pattern with lower genetic diversity in small isolated populations compared to larger populations for all three marker types, none of our measures of MHC genetic diversity estimates were significantly correlated to SNP or microsatellite genetic diversity. This is in line with a large meta-analysis (Reed and Frankham 2001) showing no clear correlation between adaptive (measured as heritability h^2^ of quantitative traits) and neutral (H_E_) genetic diversity. Arguably, it is difficult to estimate “ecological meaningful” genetic diversity by solely measuring the neutral genetic diversity. In a recent review of MHC diversity and viability in natural populations, each case study included at least some populations that had undergone a decrease in population size (i.e., like our study) (Radwan et al. 2010). A majority of these case studies showed a significant positive correlation between neutral markers with MHC AR, contrary to our study. Based on these results, Radwan et al. (2010) suggested that demographic processes rather than selection shape MHC variation in the short timescale. It is difficult to exactly predict how drift and balancing selection are shaping genetic variation at neutral and MHC loci at any given point in the history of a population subjected to reduction in population size. At large population sizes, subjected to moderate drift, it is believed that balancing selection is upholding genetic variation at the MHC, making these loci more variable than neutral loci (Takahata 1990). With lower population size, the effect of drift becomes more severe (Kimura 1983) and would eventually become the most powerful force also at loci subjected to balancing selection. How long the legacy of past balancing selection will last as “a long-term effective population size” (Takahata 1990) depends on the severity of the population size reduction and the intensity and nature of past selection. We suggest that balancing selection is the most important process affecting MHC loci at large and moderate population sizes while drift (demographic processes) is the most important when populations become really small and isolated, as indicated by our data. However, this conclusion is partly dependent on the nature of balancing selection. It has recently been suggested that a pure overdominance (i.e., heterozygote advantage) model is not powerful enough to create observed levels of diversity in large populations and thus that some form of frequency-dependent selection must have been operating (van Oosterhout, 2009). Frequency-dependent selection may help to further reduce genetic variation during bottlenecks (Ejsmond and Radwan 2011; Sutton et al. 2011).

### MHC versus SNP and microsatellite population structure

Both microsatellite and SNP markers show a similar population structure pattern with a trend for small isolated populations to be more genetically divergent than the other population categories. MHC however, does not display similar population structure as microsatellite or SNP markers (comparing F_ST_/(1 – F_ST_) correcting for geographic distance, Fig. 3). However, when removing small isolated populations from the comparison, MHC F_ST_/(1 – F_ST_) were significantly correlated with the corresponding metric from microsatellites. This is interesting as it may suggest that drift is affecting microsatellites differently from MHC in small isolated populations. Logically, there are three different possible outcomes comparing pairwise MHC F_ST_ with F_ST_ in neutral markers. MHC class II F_ST_ (or G’_ST_) can be lower (van Oosterhout et al. 2006; Fraser et al. 2009; Evans et al. 2010), equal (Hedrick 2001), or higher (Ekblom et al. 2007; Miller et al. 2010) than F_ST_ from neutral markers (microsatellite). In simulations, G_ST_ (the ratio of between-deme diversity to total diversity, equivalent to F_ST_) is lower for genes under balancing selection (including overdominance/heterozygote advantage) than for neutral genes (Schierup et al. 2000). This is suggested to be because, under balancing selection, genetic diversity within populations is maintained and also, a migrant allele (but already present in the global population) is selected for compared to a migrant neutral allele (Schierup et al. 2000). However, when new MHC alleles are selected for, the allele pool will be different in different populations, resulting in higher population structure at MHC compared to neutral loci (Spurgin and Richardson 2010). When there is local selection operating on genes within the populations, F_ST_ has been simulated to be higher than for neutral genes (Charlesworth et al. 1997). In most studies reporting a higher F_ST_ for MHC than for neutral loci (Ekblom et al. 2007; Miller et al. 2010), there are supposedly different pathogen regimes in different habitats enhancing different MHC alleles (Spurgin and Richardson 2010). It is not easy to prove that the genetic structure is lower, higher, or similar between markers but there is at least a trend for MHC F_ST_ (0.031) to be lower compared to both microsatellite F_ST_ (0.142) and SNP F_ST_ (0.232) (Fig. 3). We did not observe any private MHC alleles in the populations. However, since only MHC alleles observed from cloning and sequencing in two independent PCR reactions were used in the RSCA scoring procedure, it is possible that private MHC alleles may have gone undetected in our dataset. We still consider that the population structure is lower in MHC BLB than neutral markers. This probably means that there is a uniform selection pressure from pathogens across European black grouse population, reflecting past wider black grouse abundance in Europe. Neutral markers respond faster to genetic drift and therefore populations are genetically more divergent in microsatellites than in MHC.

Isolation by distance provides information about gene flow and the connectivity of populations. No isolation by distance was found for either microsatellites, SNPs, or MHC reflecting the relative isolation of many of the sampled populations. When analyzing only continuous populations, despite the resulting low sample size, genetic distance based on SNPs was found to be positively correlated with geographic distance, following an isolation by distance pattern (but not for MHC or microsatellites). For MHC, no isolation by distance probably reflects the action of balancing selection in the past due to widespread disease organisms. For the neutral markers, the absence of isolation by distance at least in the isolated categories may reflect the strong action of drift leading to strong genetic differences among populations, which is also highlighted by F_ST_ values. Isolated populations are likely subjected to stochastic events and therefore drift affects these populations more strongly. On the contrary, our samples from the continuous populations in Scandinavia reflect the natural dispersal of the species and genetic differences may be prominent only over larger distances (see also similar results for capercaillie in Segelbacher and Storch 2002).

### Are microsatellites and SNPs correlated?

We found that the population structure estimates for microsatellites and SNPs were similar. Microsatellites and SNP pairwise F_ST_/ (1 – F_ST_) were significantly correlated for all combinations of population categories and markers. In addition, genetic diversity measures obtained from microsatellites and SNP were positively correlated (*r* = 0.62–0.70, although not significantly so after Bonferroni correction). Moreover, microsatellite and SNPs revealed mainly the same patterns of lower genetic diversity in smaller populations compared to larger (Fig. 2a and b). As expected, the isolated population category had significantly higher genetic diversity in SNPs (percentage polymorphic loci) compared to the small isolated category, which was not evident from microsatellites. Allelic differences may be affected more rapidly in microsatellites compared to SNP markers. Alternatively, the ability of detecting effects of isolation may be dependent on the type of markers used. The slightly different patterns for SNPs and microsatellites could be interpreted as the SNPs display higher resolution than the microsatellites. Given that a simulation-based study reported that for reliably estimating genome-wide levels of variation four to 10 times more biallelic markers are necessary compared with multiallelic markers (Mariette et al. 2002; Haasl and Payseur 2011), it is somewhat surprising that our relatively low number of SNPs (21) managed to accurately reproduce the same patterns of genetic differentiation as a set of nine microsatellite loci and perhaps also with better resolution. An explanation for this may be that the SNPs were specifically selected to be polymorphic among the studied populations. Overall, a positive correlation between microsatellites and SNPs has been found in also in other studies (Väli et al. 2008). The fact that microsatellites and SNPs in this study correlates implies that these markers reflect genome-wide neutral patterns.

### Implication of our results

The divergent patterns of diversity and spatial structure on MHC and neutral markers suggest that there may be some factor that is (or has previously been) involved in maintaining MHC variation. Black grouse is similar in the MHC organization compared to chicken (Strand et al. 2007; Strand et al. unpubl. data, Wang et al. unpubl. data) and may thus show similar strong associations to diseases. In a seminatural population of red jungle fowl (i.e., the wild form of domestic chicken), it has been shown that MHC heterozygote individuals survived an infection of coccidiosis longer than homozygotes (Worley et al. 2010). It was suggested that in large outbred populations, most birds would be likely to be MHC heterozygotes and therefore susceptible genotypes are not often homozygous. In inbred and bottlenecked populations, MHC homozygosity is increased and may thus affect survival. However, it is difficult to prove that loss in MHC diversity affects the survival of populations (Radwan et al. 2010) but see Siddle et al. (2007). If MHC diversity is important for black grouse, small isolated black grouse populations may likely be more prone to extinction given the low number of only a few individuals reported in recent years. To prevent isolated populations with still large population size to follow, we suggest restoration of spatial connectivity among the isolated populations if possible and take all management options to prevent the populations from a further decline.

## Conclusions

This study is, to our knowledge, the first one in combining microsatellites, SNPs and MHC markers to gain a wider and deeper knowledge of genetic diversity in natural populations of varying degree of isolation and size. We included several populations in each population category to identify possible differences in neutral and MHC genetic diversity. We show that small isolated populations of black grouse display significant lower neutral genetic diversity than continuous populations. In addition, genetic variation within the MHC complex also seems to be lowered. The power of balancing selection acting on MHC locally may therefore not be enough to counteract genetic drift in populations that are both small and isolated. However, the pattern of lower population structure in MHC than microsatellites or SNPs, suggests that selection on MHC is still operating across European black grouse populations.

## Data Archiving

MHC allele GenBank accessions: BLB11 = GQ181215, BLB13 = JF509706, BLB14 = HQ108380, BLB16 = HQ108382, BLB18 = HQ108383, BLB21 = HQ108384, BLB22 = HQ108385, BLB23 = JF509707.

## Supporting Information

**Table S1.** Pairwise F_ST_ (below the diagonal)and D_EST_ (above the diagonal) (MHC, Microsatellites and SNPs) and distance matrices (in kilometers).

**Figure S1.** Genetic variation comparing populationcategories continuous (*n* = 5), isolated (*n* = 3), and small isolated (*n* = 3) for (a) microsatellite allelic richness (AR), (b) SNP polymorphic loci, (c) total number of different MHC alleles in populations divided by sample size (MHC/pop), (d) mean number of alleles per individual among populations (MHC/ind), (e) MHC average percent difference (APD), and (f) MHC Pi.

**Figure S2.** The number of individuals carrying aparticular MHC class II allele.

**Figure S3.** Comparisons between microsatellite and SNPmarkers.
