# Supplementary material for: Can balancing selection on MHC loci counteract genetic drift in small fragmented populations of black grouse?
Source: Ecol Evol. 2012 Feb;2(2):341–53. doi: 10.1002/ece3.86 (PMC3298947; doi:10.1002/ece3.86)
Supplement: Supplementary file 1 [file ece30002-0341-SD1.doc]

Supplementary information

**Can balancing selection on MHC loci counteract genetic drift in small fragmented populations of black grouse?**

Tanja M Strand^1^; Gernot Segelbacher^1,2^; María Quintela^1,3^; Lyngyun Xiao^1^; Tomas Axelsson^4^; and Jacob Höglund^1^

**Supplementary Table S1**. Pairwise F_ST_ (below the diagonal) and D_EST_ (above the diagonal) (MHC, Microsatellites and SNPs) and distance matrices (in kilometers). Values significantly from zero (after Bonferroni correction) are in bold. The negative F_ST_ and D_EST_ values can be read as no differentiation between pairs since they are due most probably to sampling variation. Notice that D_EST_ values for pairs with Norway are missing for the microsatellite data. Due to missing genotypes at two microsatellite loci for Norway the program SMOGD could not calculate D_EST_ values for population pairs with Norway. The differentiation index D_EST_ was for microsatellites 0.413, for SNPs 0.042 and for MHC 0.111. Notice that F_ST_ was calculated in FSTAT for microsatellites and SNPs, and in Arlequin for MHC. D_EST_ was calculated in the program SMOGD for microsatellites and SNPs but in the program SPADE for MHC. Population code in Table 1.

| **MHC F_ST_\D_EST_** | **Alps_C** | **Fin_C** | **Lat_C** | **Nor_C** | **SweJ_C** | **Eng_I** | **LH_I** | **Pol_I** | **Aus_SI** | **Ger_SI** | **Neth_SI** |
| --- | --- | --- | --- | --- | --- | --- | --- | --- | --- | --- | --- |
| Alps_C |  | 0.195 | 0.057 | 0.332 | -0.047 | 0.235 | -0.001 | 0.044 | 0.031 | -0.061 | 0.080 |
| Fin_C | 0.039 |  | -0.070 | 0.073 | 0.033 | 0.248 | 0.176 | 0.339 | 0.136 | 0.137 | 0.083 |
| Lat_C | 0.022 | -0.019 |  | 0.071 | -0.098 | 0.233 | 0.051 | 0.185 | 0.060 | -0.013 | 0.046 |
| Nor_C | 0.087 | 0.016 | 0.002 |  | 0.138 | 0.108 | 0.117 | 0.433 | 0.393 | 0.332 | 0.118 |
| SweJ_C | -0.010 | -0.005 | -0.047 | -0.008 |  | 0.183 | 0.002 | -0.019 | -0.091 | -0.074 | -0.032 |
| Eng_I | 0.043 | 0.036 | 0.043 | 0.047 | 0.020 |  | 0.316 | 0.421 | 0.386 | 0.382 | 0.205 |
| LH_I | 0.034 | 0.062 | 0.042 | 0.050 | 0.039 | 0.113 |  | 0.124 | 0.112 | -0.005 | 0.052 |
| Pol_I | 0.031 | 0.101 | 0.063 | 0.126 | -0.021 | 0.136 | 0.079 |  | 0.050 | -0.025 | 0.186 |
| Aus_SI | -0.006 | 0.024 | 0.008 | 0.088 | -0.036 | 0.065 | 0.076 | 0.057 |  | -0.020 | 0.049 |
| Ger_SI | -0.019 | 0.017 | -0.018 | 0.069 | -0.023 | 0.082 | 0.023 | -0.006 | 0.004 |  | 0.097 |
| Neth_SI | 0.016 | 0.013 | -0.005 | 0.017 | -0.036 | 0.041 | 0.034 | 0.049 | -0.002 | 0.013 |  |
|  |  |  |  |  |  |  |  |  |  |  |  |
| **Microsatellites F_ST_\D_EST_** | **Alps_C** | **Fin_C** | **Lat_C** | **Nor_C** | **SweJ_C** | **Eng_I** | **LH_I** | **Pol_I** | **Aus_SI** | **Ger_SI** | **Neth_SI** |
| Alps_C |  | 0.438 | 0.077 | - | 0.072 | 0.288 | 0.364 | 0.224 | 0.275 | 0.201 | 0.375 |
| Fin_C | **0.131** |  | 0.232 | - | 0.281 | 0.525 | 0.262 | 0.351 | 0.470 | 0.432 | 0.518 |
| Lat_C | **0.032** | **0.079** |  | - | 0.010 | 0.282 | 0.166 | 0.119 | 0.332 | 0.146 | 0.360 |
| Nor_C | **0.108** | **0.094** | **0.057** |  | - | - | - | - | - | - | - |
| SweJ_C | **0.049** | **0.094** | **0.017** | **0.087** |  | 0.243 | 0.205 | 0.308 | 0.314 | 0.212 | 0.287 |
| Eng_I | **0.120** | **0.207** | **0.135** | **0.137** | **0.133** |  | 0.352 | 0.444 | 0.440 | 0.531 | 0.551 |
| LH_I | **0.148** | **0.136** | **0.095** | **0.121** | **0.105** | **0.220** |  | 0.240 | 0.548 | 0.396 | 0.223 |
| Pol_I | **0.107** | **0.136** | **0.072** | **0.113** | **0.144** | **0.228** | **0.153** |  | 0.335 | 0.151 | 0.537 |
| Aus_SI | **0.141** | **0.214** | **0.147** | **0.175** | **0.160** | **0.244** | **0.249** | **0.202** |  | 0.199 | 0.513 |
| Ger_SI | **0.075** | **0.145** | **0.082** | **0.128** | **0.095** | **0.220** | **0.172** | **0.102** | **0.110** |  | 0.300 |
| Neth_SI | **0.192** | **0.257** | **0.202** | **0.234** | **0.188** | **0.343** | **0.160** | **0.288** | **0.309** | **0.213** |  |
|  |  |  |  |  |  |  |  |  |  |  |  |
| **SNP F_ST_\D_EST_** | **Alps_C** | **Fin_C** | **Lat_C** | **Nor_C** | **SweJ_C** | **Eng_I** | **LH_I** | **Pol_I** | **Aus_SI** | **Ger_SI** | **Neth_SI** |
| Alps_C |  | 0.000 | 0.001 | 0.000 | 0.000 | 0.000 | 0.000 | 0.000 | 0.000 | 0.000 | 0.000 |
| Fin_C | **0.158** |  | 0.431 | 0.000 | 0.041 | 0.000 | 0.000 | 0.000 | 0.000 | 0.000 | 0.000 |
| Lat_C | 0.094 | 0.001 |  | 0.038 | 0.036 | 0.000 | 0.005 | 0.001 | 0.000 | 0.000 | 0.000 |
| Nor_C | **0.101** | **0.080** | 0.045 |  | 0.016 | 0.000 | 0.000 | 0.000 | 0.000 | 0.000 | 0.000 |
| SweJ_C | **0.134** | 0.045 | 0.065 | 0.056 |  | 0.000 | 0.001 | 0.000 | 0.002 | 0.000 | 0.000 |
| Eng_I | **0.425** | **0.295** | **0.293** | **0.312** | **0.244** |  | 0.000 | 0.000 | 0.000 | 0.000 | 0.000 |
| LH_I | **0.085** | **0.102** | 0.092 | **0.098** | 0.096 | **0.312** |  | 0.000 | 0.000 | 0.000 | 0.000 |
| Pol_I | **0.254** | **0.150** | 0.134 | **0.246** | **0.280** | **0.425** | **0.167** |  | 0.000 | 0.000 | 0.000 |
| Aus_SI | **0.153** | **0.207** | 0.187 | **0.212** | **0.127** | **0.393** | **0.118** | **0.327** |  | 0.000 | 0.000 |
| Ger_SI | **0.312** | **0.233** | **0.245** | **0.350** | **0.309** | **0.456** | **0.214** | **0.214** | **0.227** |  | 0.000 |
| Neth_SI | **0.277** | **0.256** | **0.224** | **0.277** | **0.335** | **0.559** | **0.149** | **0.294** | **0.327** | **0.328** |  |
|  |  |  |  |  |  |  |  |  |  |  |  |
| **Distance (km)** | **AlpsC** | **FinC** | **LatC** | **NorC** | **SweJC** | **EngI** | **LHI** | **PolI** | **AusSI** | **GerSI** | **Neth_SI** |
| Alps_C |  |  |  |  |  |  |  |  |  |  |  |
| Fin_C | 2020 |  |  |  |  |  |  |  |  |  |  |
| Lat_C | 1470 | 590 |  |  |  |  |  |  |  |  |  |
| Nor_C | 1260 | 1080 | 850 |  |  |  |  |  |  |  |  |
| SweJ_C | 1740 | 640 | 710 | 530 |  |  |  |  |  |  |  |
| Eng_I | 1220 | 1820 | 1550 | 750 | 1220 |  |  |  |  |  |  |
| LH_I | 712 | 1390 | 910 | 580 | 1010 | 810 |  |  |  |  |  |
| Pol_I | 620 | 1430 | 870 | 950 | 1270 | 1290 | 480 |  |  |  |  |
| Aus_SI | 470 | 1660 | 1100 | 1160 | 1490 | 1380 | 630 | 230 |  |  |  |
| Ger_SI | 430 | 1620 | 1120 | 870 | 1310 | 950 | 290 | 400 | 420 |  |  |
| Neth_SI | 675 | 1590 | 1170 | 660 | 1160 | 630 | 240 | 670 | 740 | 320 |  |

**Supplementary Figure S1.** Genetic variation comparing population categories continuous (n=5), isolated (n=3) and small isolated (n=3) for a) microsatellite allelic richness (AR), b) SNP polymorphic loci, c) total number of different MHC alleles in populations divided by sample size (MHC/pop), d) mean number of alleles per individual among populations (MHC/ind), e) MHC average percent difference (APD) and f) MHC Pi.


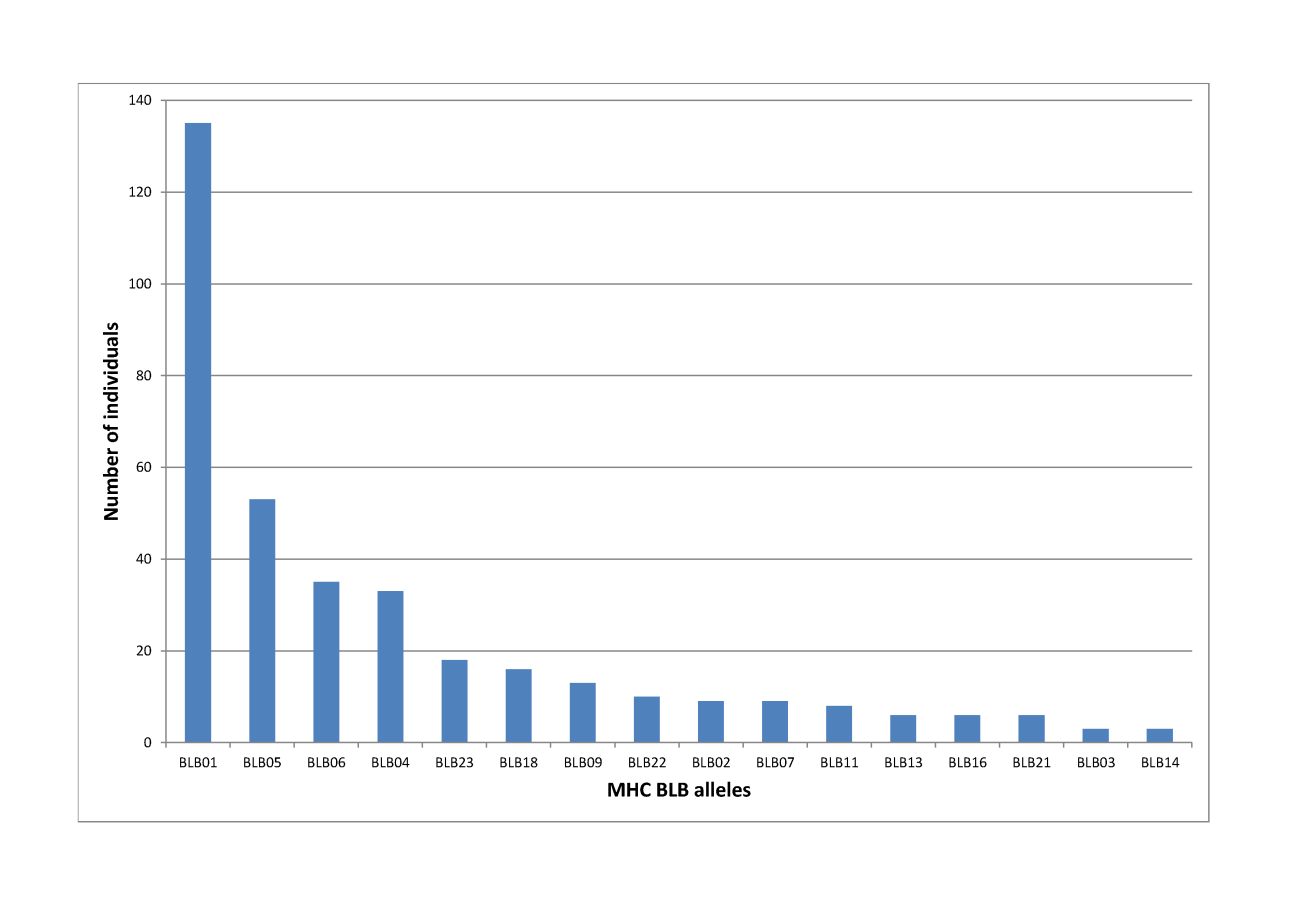


**Supplementary Figure S2**. The number of individuals carrying a particular MHC class II allele.

**Supplementary Figure S3**. Comparisons between microsatellite and SNP markers. (a) plot displaying the significant correlation between microsatellite and SNP heterozygosity (Pearson statistics, n=11) and (b) pairwise microsatellite F_ST_/(1-F_ST_) and SNP F_ST_/(1-F_ST_) (n=11) (significance of correlation was tested with partial mantel test and Mantel test in R (10 000 permutations)).
